# Cuckoo Search Approach for Parameter Identification of an Activated Sludge Process

**DOI:** 10.1155/2018/3476851

**Published:** 2018-01-28

**Authors:** Intissar Khoja, Taoufik Ladhari, Faouzi M'sahli, Anis Sakly

**Affiliations:** Industrial Systems Study and Renewable Energy Unit, National Engineering School of Monastir, University of Monastir, Ibn El Jazzar Street, Skanes, 5019 Monastir, Tunisia

## Abstract

A parameter identification problem for a hybrid model is presented. The latter describes the operation of an activated sludge process used for waste water treatment. Parameter identification problem can be considered as an optimization one by minimizing the error between simulation and experimental data. One of the new and promising metaheuristic methods for solving similar mathematical problem is Cuckoo Search Algorithm. It is inspired by the parasitic brood behavior of cuckoo species. To confirm the effectiveness and the efficiency of the proposed algorithm, simulation results will be compared with other algorithms, firstly, with a classical method which is the Nelder-Mead algorithm and, secondly, with intelligent methods such as Genetic Algorithm and Particle Swarm Optimization approaches.

## 1. Introduction

The waste water coming from domestic or industrial sources may contain many unknown components which can endanger the lives of human beings, animals, and plants. Waste water treatment process allows exposing and eliminating these toxic materials in order to prevent their discharge into the environment.

This treatment may be divided into two main tasks: a physical one (preliminary/primary treatment) to remove solid materials and a biological one (secondary treatment) to remove both dissolved and particulate organic materials. The most common process for the second operation is the activated sludge process. The latter is based on the cultivation of microorganisms' population that consummates the pollutants existing in the wastewater as food source [1].

In order to obtain an optimal purifying performance and to enable the users to better manage, scientifically and technically, their wastewater treatment systems many modeling and controlling strategies have been established.

In 1982, the International Water Association (IWA) created a group that worked on mathematical modeling for design and implementation of activated sludge processes. Their primary purpose was to develop a realistic model that describes efficiently the operation of these systems. The result was the activated sludge model number 1 (ASM1). It is, in fact a model that simulates within an activated sludge system, phenomena, such as carbon oxidation, nitrification, and denitrification by quantifying the kinetics and stoichiometry of each reaction. This model is based on the simulation of the biomass growth as the main engine of the degradation process [2]. Thereafter, many other models had appeared. In 1995, the ASM2 had been published. It included the elimination of nitrogen combined with biological phosphorus removal. However, this process was unclear. For this reason, it was replaced by the ASM2d model which included the combined denitrification.

In 1998, the working group decided to develop a new model which is the ASM3 in order to create a simple tool for future generations of models [3]. Unfortunately, all these models proved to be complicated and highly nonlinear which required creating more simpler and intelligible models [4, 5].

Parameter identification presents a prerequisite to accomplish an effective control plan and respond to the changes in both the operating conditions and the wastewater characteristics within the treatment process.

Over the years many parameter identification approaches have been studied. They can be classified into two categories: classical methods like linearized maximum likelihood [6], extended Kalman filter [7], the calculus of state variables sensibilities [8], subspace method [9], the Nelder-Mead method [4, 5], recursive prediction error method [10], the minimization of an Euclidian-distance criterion [11], and so on and intelligent methods like Genetic Algorithm [12], Particle Swarm Optimization [13], and so on.

By outperforming the classical methods (especially for the nonlinear, stochastic, complex, and multidimensional optimization problems), the intelligent ones become more and more considered in different scientific fields [14]. One of the recent techniques is the Cuckoo Search Algorithm (CSA). It is based on the interesting breeding behavior of certain bird species called cuckoo. Up to now, this algorithm has been investigated to work out a variety of optimization problems [15–19]. Thanks to its efficiency and robustness, it has been specially considered for the parameter identification problem [20–23].

In this paper, Cuckoo Search Algorithm will be adapted and applied to parameter identification of a hybrid linear model which describes the dynamics of an activated sludge process for wastewater treatment [5].

In order to prove the CSA's effectiveness in performing the identification exercise, simulation results will be compared with other techniques: firstly, a classical one which is the Nelder-Mead method and secondly, intelligent ones which are the GA and PSO, not forgetting to mention the real systems measurements. The remaining sections are organized as follows: in Section 2 a presentation of the activated sludge's modeling problem is detailed. After that, an overview of the different algorithms' concepts is stated in Section 3. Last but not least, Section 4 provides the simulation results as well as the comparison between the different proposed approaches. Finally, the paper ends with a conclusion.

## 2. Process Presentation

### 2.1. Description

**Figure 1:**
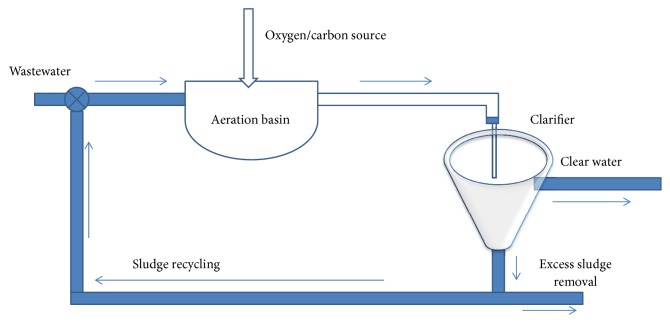
Activated sludge process.

At its basic level, the activated sludge process (ASP) is composed of two interconnected tanks which are a bioreactor and a settler as shown in Figure 1. The first one presents a cultivation environment of specific bacteria species (heterotrophic and autotrophic population). Their role consists of degrading the organic substrates (phosphorus, nitrogenous, and carbonaceous pollutants) founded in the effluent. As a result, they transform into activated sludge. The second one separates between the treated water and the formed sludge. A part of this latter will be recycled back to the bioreactor and the other will be rejected. The pure water will be evacuated to be used later.

The bioreaction of ASP takes place, essentially, in the aerator. It can be divided into two main phases. The first one is the nitrification/aerobic stage. It consists in providing an oxygen source for the bacteria nutrition in order to eliminate the nitrate and carbon substrates. These microorganisms will agglomerate into flocks and produce the sludge. The second is the denitrification/anoxic stage. It consists in shutting down the provision of oxygen and providing an external carbon source. However, the microorganisms pursue the consumption of the remaining oxygen to continue the degradation procedure until its total disappearance. This presents a transitional phase which is usually very short and belongs to the aerobic phase. The switching between the phases is ensured by the change of value for the oxygen transfer coefficient (*k*_*La*_). In the aeration period, its value is different from 0. However, in the anoxic period it is equal to 0. Hence, the aeration procedure can be qualified as discontinuous.

As a powerful tool for water purification, the ASP needs to be introduced by a mathematical model that will describe all aspects and phenomena that take place. As mentioned before, many models have been designed for this plant, trying to offer, on the one hand, a precise and simple understanding of this complex process and an efficient presentation for real-time use and control strategies on the other hand.

The examined ASP is a pilot unit installed in the Engineering Laboratory of Environmental Processes (ELEP) of the National Institution of Applied Sciences (NIAS) in Toulouse-France.

This unit presents the basic structure of activated sludge. The only available data are the nitrate, the ammonium, and the oxygen concentrations which make the creation of more simplified models a necessity. Numerous reduction methods have been applied to achieve this goal. The considered method in this paper is based on some biochemical considerations (observation of variables' behavior and their influence over kinetics reactions and other variables) made on a reference model inspired from the ASM1 [4] as well as the adjustment of the reduced-order model to ensure the conservation of controllability and observability properties [4, 5].

### 2.2. Linear Model

Up to date, linear models are the most considered and devoted mathematical models in the different automation areas: estimation, control, diagnostic, and so on. Diversity of methods and theories has been developed for these aims, in contrast with the limited numbers of studies for the other system types (bilinear, nonlinear, etc.).

Researchers are invited to develop a linear presentation for the activated sludge system. Despite their efficiency, designed linear models for ASP are few. In [24] authors have developed a linear model formed of two submodels: one for the aerobic phase and the other for the anoxic one. It is composed of eight state variables. After that in [25] authors have proposed a linearized model for the ASM1 model but it is too complicated.

The main interest in realizing a linear presentation for a complex model is the choice of the corresponding method, taken into consideration the preservation of the model's physical meaning. The proposed ASP's linear model is the one developed in [5, 26]. It is obtained by applying, for a reduced-order nonlinear model [4], the Taylor Series Expansion method around a nominal trajectory given the constant alternating behavior of the process. The obtained linear model contains two submodels: the first one presents the aerobic phase and the second one presents the anoxic phase. The switch between these models is organized by the *k*_*La*_ coefficient. Therefore, in the aeration phase, the model has four state variables which are the substrate, the nitrite, the ammonium, and the oxygen. For the other phase, it has only three state variables since the oxygen variable will be eliminated. The general model can be presented by the following equation:

(1) $$\begin{array}{c} \overset{.}{x}\left(\;t\;\right)=A_{a,b}x\left(\;t\;\right)+B_{a,b}u\left(\;t\;\right), \end{array}$$

where *x*(*t*) = [*S*_s_  *S*_NO_3__  *S*_NH_4__  *S*_O_2__] is the state vector and *u*(*t*) = [*S*_sc_  *S*_sin_  *S*_NH_4in__1] presents the input vector where *S*_sc_ can be considered as the control variable. *S*_sin_ and *S*_NH_4_in_ are hardly measured online and they are more considered as additive disturbances. 1 completes the input vector. *A*_*a*_, *B*_*a*_ are the matrices for the aerobic stage whereas *A*_*b*_, *B*_*b*_ are the matrices for the anoxic stage. *β*_*i*_ presents the specific parameters of the linear model. The other variables are defined in “Variables' Definition.”

(2) Aa=−Ds+Dc−β1YH0000−Ds+Dcβ40−iNBMβ10−β4−Ds+Dc0−1−YHYHβ10−4.57β4−kLa−Ds+Dc,Ba=DcDs0β7000β500Ds−β5+β6000−4.57β5+kLaSO2sat,Ab=−Ds+Dc−β3YH−β2YH+β8001−YHβ22.86YH−Ds+Dc−1−YHβ22.86YH00−iNBMβ3−iNBMβ2−Ds+Dc00000,Bb=DcDs0β9000000Dsβ60000.

## 3. Methods

### 3.1. Cuckoo Search Algorithm

Cuckoo Search Algorithm (CSA) is a new nature-inspired optimization method. It was introduced in 2009 by Yang and Deb. This metaheuristic is built on two basic concepts: firstly the breeding behavior of some special birds species (cuckoo) and secondly the characteristics of Lévy flights of some fruit flies and birds.

#### 3.1.1. Cuckoos' Behavior

The cuckoo is a discreet bird of medium size and its captivating voice marks the beginning of a beautiful season. It is well known by its fascinating reproductive strategy. The brood parasitism of some species is the most studied and discussed.

The cuckoo female puts one or several eggs in others species' nests, previously observed. The aim is to ensure a smooth transition to the following generation by leaving the host birds guided by their natural instinct of breeding, hatching, and bringing food to the small cuckoos.

In order to increase the surviving chance of the new cuckoo, the female gobbles an egg in the host's nest, before laying its own. Some host species may have conflicts with the intruder cuckoo. When host birds discover the presence of egg that does not belong to them, thanks, for example, to a sensitive skin area under their bellies, they get rid of it or abandon the nest by constructing a new one elsewhere. Some types of cuckoos like the Tapera are very intelligent. They have developed the ability to imitate the host's eggs in color and shape. This reduces the probability of their eggs being abandoned and thereby increasing their reproductive capacity.

#### 3.1.2. Lévy Flights

Lévy flights, named by the French mathematician Paul Lévy, represents a mathematical model for random walks characterized by their step lengths which follow a power law written in the following form:

(3) $$\begin{array}{c} y=l^{-\lambda }, \end{array}$$

where *l* presents the flight length and *λ* presents the variance. Since 1≺*λ*≺3, *y* has an infinite variance.

Finding a suitable host nest plays a key role in the success of cuckoo's reproduction strategy. Generally, the nest's search plan is similar to food search, given that, in nature, animals seek their food randomly or quasirandomly. They choose trajectories or directions that can be described by certain mathematical equations.

**Figure 2:**
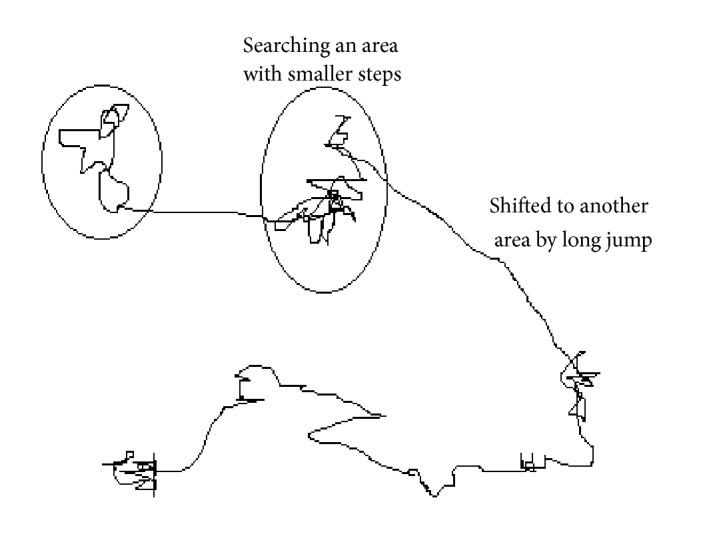
Example of Lévy flight in 2-dimensional plan.

Different studies have shown that the flight behavior of many birds and insects has the same characteristics of the typical Lévy flights. Recent one views that fruit flies or “*Drosophila melanogaster*” dig into their landscape using a series of straight trajectories punctuated by a sharp turn of 90°, which leads to a search pattern of Lévy flight style. This model is commonly represented by small random steps followed by large jumps as shown in Figure 2.

Such behavior combined with the cuckoo breeding behavior forms an effective metaheuristic method for optimization problems. Being compared to other metaheuristic approaches, the long jumps reinforce, significantly, the exploration ability of cuckoos in the search space especially needed for solving multimodal nonlinear problems [27].

#### 3.1.3. Algorithm

The CSA is based on the following rules:

- Each cuckoo chooses a nest randomly in which it lays one egg at a time.
- The best nest with the highest eggs' quality can pass to the new generation.
- The number of the host nests is fixed, and the cuckoo's posed egg can be discovered by the host bird according to a probability *P*_*a*_ ∈ [0, 1].

In the case of discovering the cuckoo's eggs, the host bird can destroy them or abandon its nest. In the two scenarios, a new nest will be developed with the probability *P*_*a*_ for a fixed number of nests.

**Figure 3:**
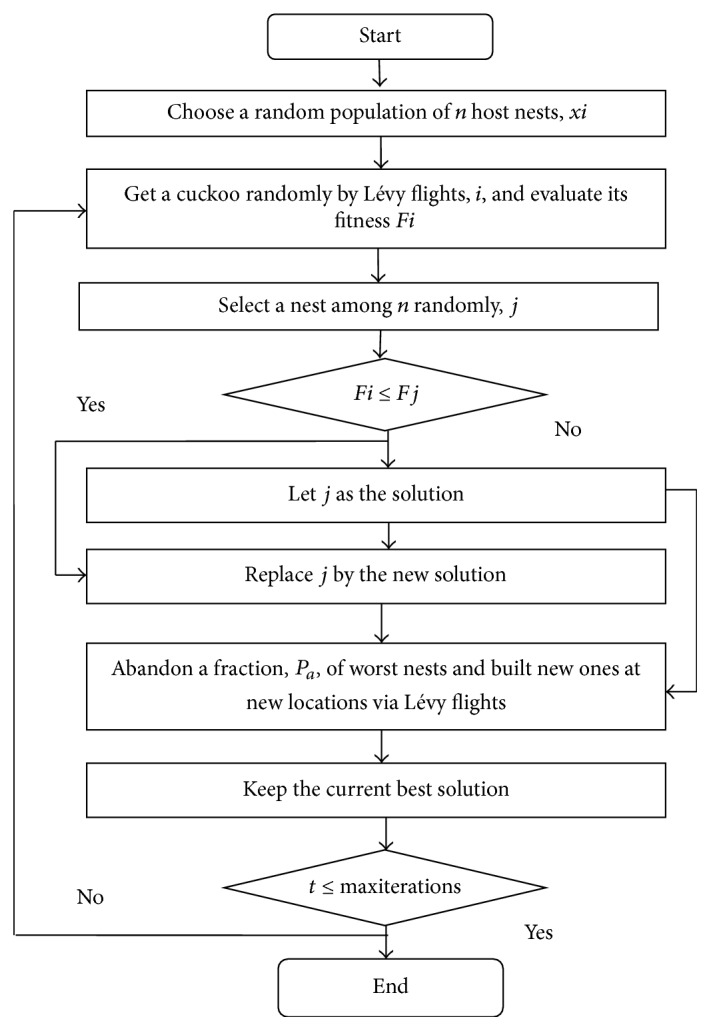
Flowchart of CSA.

Referring to these ideas, the CSA can be summarized in the flowchart of Figure 3.

In order to generate a new solution *x*^(*t* + 1)^ for a cuckoo *i*, a Lévy flight is executed as dictated by this expression:

(4) $$\begin{array}{c} x_{i}^{\left(t+1\right)}=x_{i}^{t}+\alpha ⊕\text{Lévy}\left(\;\lambda \;\right), \end{array}$$

where *x*_*i*_^*t*^ presents samples (eggs), *t* is the number of iteration, *i* is the sample number, and *α*≻0 is the step size. It is important to tune this value in order to get the desired step size controlled by the problem's constraints. The product ⊕ means the entry-wise multiplication. Lévy(*λ*) is calculated from Lévy distribution as follows:

(5) $$\begin{array}{c} \text{Lévy}\left(\;\lambda \;\right)\approx y=l^{-\lambda }. \end{array}$$

The Lévy distribution can be simplified by the following equation:

(6) $$\begin{array}{c} \alpha ⊕\text{Lévy}\left(\;\lambda \;\right)\approx k\times \left(\;\frac{u}{{\left(\left|v\right|\right)}^{1/\beta }}\;\right)\left(\;x_{best}-x_{i}\;\right), \end{array}$$

where *k* is the Lévy multiplication coefficient fixed by users, *β* = 1.5, and *u* and *v* are deducted from the normal distribution curves.

The main considered method for solving parameter identification problem is CSA but in order to prove its efficiency this latter will be compared with other methods which are briefly mentioned as follows.

### 3.2. Nelder-Mead Algorithm

The Nelder-Mead algorithm (NM) called also simplex algorithm has been suggested by Spendley, Himsworth, and Hext in 1962. Then it has been developed in 1965 by Nelder and Mead. It is a well-known classical technique for multidimensional unconstrained optimization issues. The elimination of derivatives calculation made it a very useful tool for parameters identification and other statistical problems dealing with nonsmooth or discontinuous functions.

It is necessary to separate this technique from the known Dantzig's simplex method for linear models. It is a simple algorithm that is quite easy to handle. It is founded that, on a simplex which is a geometrical structure composed of (*N* + 1) points in *N*-dimension space, segments will be designed to connect them; as a result, polygonal surfaces will be constructed such as a segment on a line, a triangle in two-dimensional space, and a tetrahedron in three dimension space [28].

### 3.3. Genetic Algorithm

The Genetic Algorithm (GA) is an optimum searching algorithm. It is inspired by the natural evolution process. It manipulates a fixed-size population, whose elements are named chromosomes. They present no other than the candidate solutions of the considered problem. These individuals work out to adapt to their living environment [29].

The main steps in the GA are the evolutionary operators which are as follows:

- Selection: the most fitted candidate of the population will remain genetically unchanged and passed on to the following generation. The use of this operator will guarantee the permanent existence of the best solution in the future generations.
- Crossover: it manipulates the chromosome structure by fusing the genetic information of two individuals (parents) so as to produce two new ones (children).
- Mutation: this genetic operator allows randomly the introduction of some modifications on the chromosomes which will enhance the genetic diversity from one generation to the next. The aim of mutation is to avoid the local optima.

### 3.4. Particle Swarm Optimization

The Particle Swarm Optimization (PSO) presents an evolutionary computation method to solve specially optimization problem. It is based on a simple concept.

The PSO algorithm chooses randomly a population of fixed size called “swarm.” It contains the candidate solutions of the considered problem named “particles.” These “particles” fly over a multidimensional space to locate their best position.

Each particle is associated firstly with an evaluation value determined by calculating the fitness function and secondly with a velocity that rules its motion. It also has a small memory that allows it to memorize its best-visited position (local optimum) and the best-visited position by the population (global optimum) obtained by it fascinating capacity to communicate with the other particles. Depending on this cooperation between the particles within the swarm, they will adapt a tendency: first, of their motivation to return to their optimal achieved solution and second, of the link with solutions achieved by their neighbors. So, the whole swarm will eventually reach the global optimal solution [30].

The particle movement is influenced by three components:

- An inertial component: the particle tends to follow its current direction.
- A memory component: the particle tends to go back to the best position it has ever visited.
- A social component: the particle tends to rely on the experience of its neighbors. So, it tries to head toward the best position achieved by them.

## 4. Simulation Results and Discussion

The parameter identification procedure for mathematical models which describe the biological processes must take into consideration two important facts: first the high complexity of the models (big number of state variables and parameters) and second the small quantity and sometime the poor quality of the available real measurements.

The role of parameter identification is to determine the model's parameters from a set of input-output measures. This task is ensured by comparing this set with the one obtained from the estimated model using a mathematical function named objective function.

In this paper, we are interested in the offline parameter identification and the Mean Square Error (MSE) is selected as the objective function called also fitness. It is given by the following expression:

(7) $$\begin{array}{c} MSE=\left(\;\frac{1}{n}\;\right)\overset{n}{\underset{i=1}{\sum }}e^{2}=\left(\;\frac{1}{n}\;\right)\overset{n}{\underset{i=1}{\sum }}{\left(\;\hat{Y_{i}}-Y_{i}\;\right)}^{2} \end{array}$$

with $\hat{Y_{i}}$ and *Y*_*i*_ being, respectively, the estimated and the measured responses of the system at each sample time *i*, (*i* = 1,…, *n*).

*e* denotes the error between these responses and *n* is the number of samples.

The real measurements are obtained from two different experiments whose duration is about 6 hours divided by a sampling period of 20 minutes under different airing conditions. One of them has been chosen for the identification procedure. The only measured state variables as we have mentioned before are *S*_NO_3__, *S*_NH_4__, and *S*_O_2__.

Taking into account the slow operating mode (limited number of samples), the parameter identification procedure will be a bit tough which makes the use of the pseudomeasurements a necessity. The new sample time is equal to 1 minute.

Our main purpose is to identify the specific parameters of the reduced linear model describing the ASP using CSA. Gather these parameters to form the following vector: $\theta =\left[\begin{array}{ccccccccc} \beta_{1} & \beta_{2} & \beta_{3} & \beta_{4} & \beta_{5} & \beta_{6} & \beta_{7} & \beta_{8} & \beta_{9} \end{array}\right]$, where each parameter corresponds to one dimension of the problem. The identification operation is built on two stages: one for the aerobic phase and the other for the anoxic one.

In the application of metaheuristic approaches, many characteristic parameters must be taken into account. They can vary from one situation to another. The unfit choice may lead the algorithm to easily fall in local optima or diverge completely.

After doing various tests, the parameters of the three intelligent algorithms have been chosen in Table 1. It is noteworthy that these algorithms can achieve a different result if another set of parameters have been applied.

The obtained results from the different approaches under identical conditions are presented in Table 2. Focusing on the intelligent ones, all these algorithms have been run five times given their random behavior with a population of 10, respectively, chromosomes, birds, and nests for 100 iterations.

According to Table 2, even though the identified parameters with different methods are not identical, they have the same order of magnitude. This numerical difference can be originated from the simplifications made on the linearized model, the choice of the objective function and the algorithms' parameters. For each method, an equivalent model is obtained, although it is different from the others.

**Figure 4:**
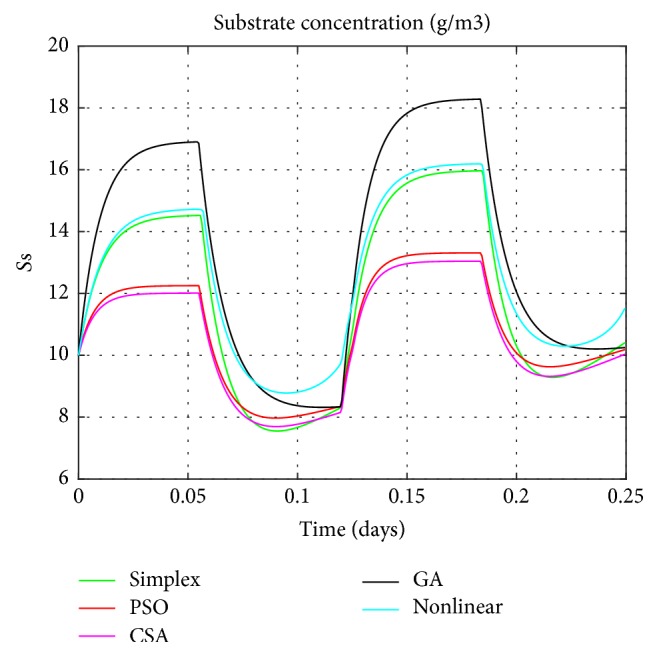
Substrate concentration.

**Figure 5:**
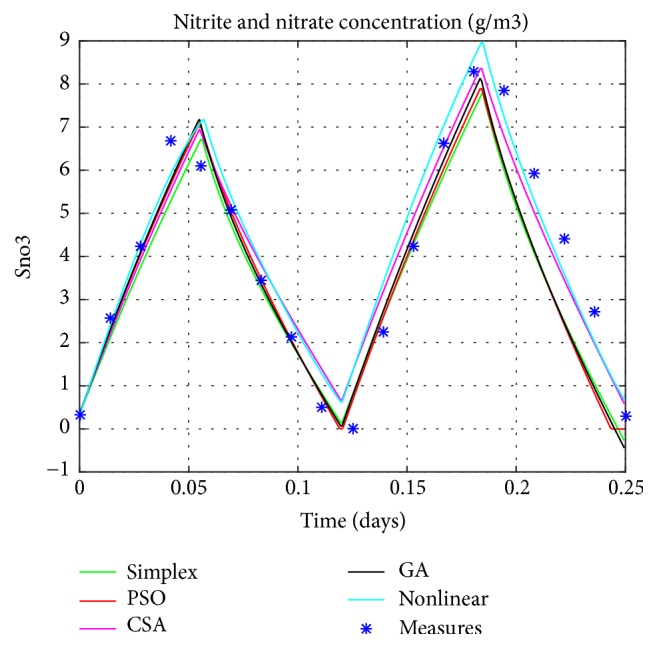
Nitrogen concentration as nitrate and nitrite.

**Figure 6:**
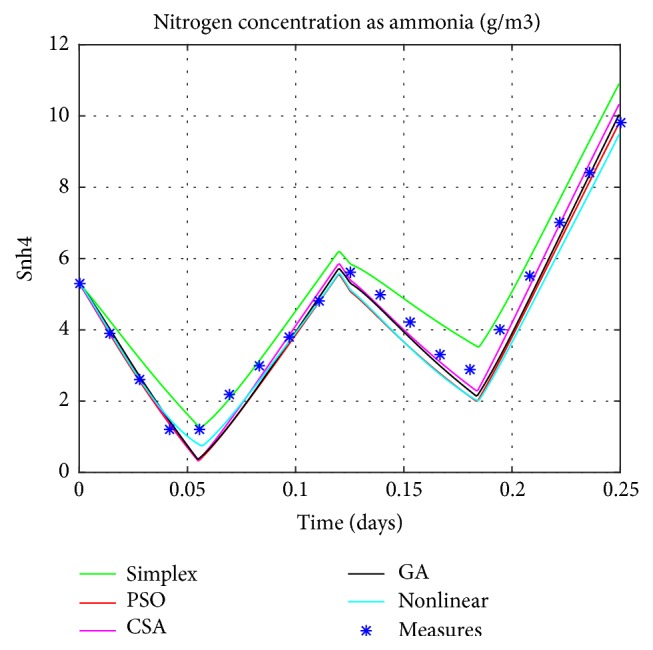
Nitrogen concentration as ammonia.

**Figure 7:**
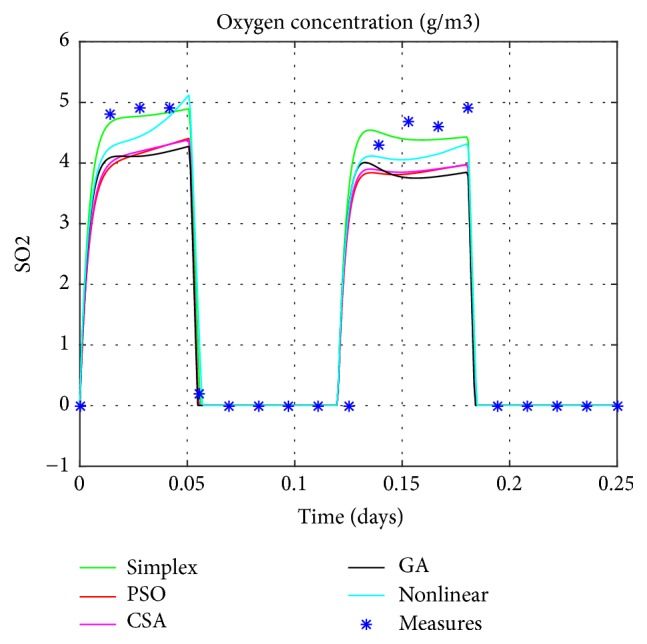
Oxygen concentration.

Graphical comparisons usually illustrate the existence or the absence of systematic deviations between real measurements and model predictions. One of the most significant criteria for the adequacy of a model is the quantitative measure of the differences between measured and calculated values. Based on the different techniques, the estimated values of parameters will be explored in the simulation of the linear model. The resulting model's responses will be compared to the real ones as exposed in Figures 4, 5, 6, and 7.

The obtained figures (Figures 4, 5, 6, and 7) show 6 different lines, 4 of them present the estimated linear model with distinctive techniques (Simplex, GA, PSO, and CSA), the fifth is the nonlinear model, and finally, the crosses show the real data performance (only for the nitrite, the ammonia, and the oxygen). These figures illustrate that the performance of the estimated model by the CSA exceeds all the others, in predicting successfully the variations of the real system responses. As a result it provides a more precise presentation so close to the original one which is the nonlinear model despite few differences specially noticed for the oxygen concentration.

This fact is also illustrated by comparing, the outputs of the estimated linear models to both the outputs of the nonlinear model (MSE1) and the experimental measures (MSE2). The Mean Square Error is used as a means of evaluation for the various considered techniques as mentioned in the Table 3.

Setting the CSA's results against the ones obtained by conventional method (Nelder-Mead method) or intelligent techniques (GA and PSO) confirms the ability of this technique to provide a simulated model that has the same dynamic as the real one. Unlike the CSA which is a generic and robust technique, the other ones (GA and PSO) require fine-tuning of their parameters for a particular problem. For example, with the same set of algorithm parameters, GA or PSO might have achieved better results for another kind of problem.

In the CSA, there are only three parameters (population size, number of iterations, and switching parameter *P*_*a*_) that determine the algorithm's effectiveness. The most important one is *P*_*a*_ which essentially rules the balance of randomization, the elitism, and the local search. Hence, the justification of the CSA competence is that there exist fewer parameters to be fine-tuned compared to GA and PSO. Thus, an efficient exploration of the search space can be easily achieved which will lead to a more efficient algorithm that can escape local optima and quickly converge toward the best solution.

It can be concluded that the Cuckoo Search Algorithm presents a fast, simple, and reduced-number-parameters method which makes it more useful than the others.

## 5. Conclusion

This paper proposes a parameter identification approach built on CSA for an activated sludge wastewater treatment process. It provides a review of the early considered method and proves its efficiency by comparing its performance with other methods classical or intelligent all along with the experimental data. Simulation results illustrate the ability of the CSA to identify the system parameter values with high precision. Hence it provides a valuable outcome to be investigated in the control strategies. This work can be carried forward by investigating the obtained model in state estimation problem in order to develop a good control strategy on this process.

## Figures and Tables

**Table 1:** Algorithms' parameters.

Parameter	Value		
	CSA	GA	PSO
Number of individuals	10	10	10
Number of generations	100	100	100
Crossover probability	-	0.9	-
Mutation probability	-	0.1	-
Selection probability	-	0.5	-
Inertia weight (*w*)	-	-	0.4 ≤ *w* ≤ 0.9
Acceleration coefficient (*c*1)	-	-	2
Acceleration coefficient (*c*2)	-	-	2
The probability (*Pa*)	0.25	-	-

**Table 2:** Identified parameters.

Parameters	Calculated values	Simplex	GA	PSO	CSA
*β*_1_	95.81	63.81	66.1065	88.36	90.96
*β*_2_	34.50	35.03	16.91	25.1149	18.4952
*β*_3_	57.48	47.0	49.9476	54.1187	48.9822
*β*_4_	6.92	5.43	6.1746	6.62	5.05
*β*_5_	108.71	101.5	112.3848	109.84	111.2
*β*_6_	78.88	53.8	60.3961	59.1801	57.7630
*β*_7_	1516.1	990.2	1291.492	1229.48	1244.64
*β*_8_	14.10	15.7	19.1219	21.2930	15.9814
*β*_9_	258.49	186.1	187.9578	265.1892	194.9514

**Table 3:** Identified parameters.

	Simplex	GA	PSO	CSA
MSE1	8.1205*e* − 04	4.3547*e* − 04	3.9683*e* − 04	2.117*e* − 04
MSE2	0.2206	0.1843	0.1723	0.0870

## Variables' Definition

*S*_*S*_: Biodegradable substrate concentration
*S*_NO_3__: Nitrate and nitrite nitrogen concentration
*S*_NH_4__: Ammonium/ammonia nitrogen concentration
*S*_O_2__: Oxygen concentration
*D*_*s*_: Dilution rate of the purge
*D*_c_: Dilution rate of carbon
*S*_sc_: Carbon concentration
*Y*_*H*_: Heterotrophic yield coefficient
*i*_NBM_: Mass of nitrogen in the biomass
*S*_O_2sat__: Oxygen saturation concentration
*k*_*La*_: Oxygen transfer coefficient
*S*_sin_: Biodegradable substrate initial concentration
*S*_NH_4_in_: Ammonium/ammonia nitrogen initial concertation.
